# Profiling the Site of Protein CoAlation and Coenzyme A Stabilization Interactions

**DOI:** 10.3390/antiox11071362

**Published:** 2022-07-14

**Authors:** Maria-Armineh Tossounian, Maria Baczynska, William Dalton, Charlie Newell, Yilin Ma, Sayoni Das, Jonathan Alexis Semelak, Dario Ariel Estrin, Valeriy Filonenko, Madia Trujillo, Sew Yeu Peak-Chew, Mark Skehel, Franca Fraternali, Christine Orengo, Ivan Gout

**Affiliations:** 1 Department of Structural and Molecular Biology, University College London, London WC1E 6BT, UK; m.tossounian@ucl.ac.uk (M.-A.T.); mb01554@surrey.ac.uk (M.B.); w.dalton@ucl.ac.uk (W.D.); charlie.newell.21@ucl.ac.uk (C.N.); y.ma.16@alumni.ucl.ac.uk (Y.M.); sayoni.das.12@alumni.ucl.ac.uk (S.D.); c.orengo@ucl.ac.uk (C.O.); 2 Departmento de Química Inorgánica Analítica y Química Física, INQUIMAE-CONICET, Universidad de Buenos Aires, Buenos Aires C1428EHA, Argentina; jsemelak@qi.fcen.uba.ar (J.A.S.); dario@qi.fcen.uba.ar (D.A.E.); 3 Institute of Molecular Biology and Genetics, National Academy of Sciences of Ukraine, 03680 Kyiv, Ukraine; filonenko@imbg.org.ua; 4 Departamento de Bioquímica, Facultad de Medicina, Universidad de la República, Montevideo 11800, Uruguay; madiat@fmed.edu.uy; 5 Centro de Investigaciones Biomédicas (CEINBIO), Universidad de la República, Montevideo 11800, Uruguay; 6 MRC Laboratory of Molecular Biology, Cambridge Biomedical Campus, Cambridge CB2 0QH, UK; spc@mrc-lmb.cam.ac.uk; 7 The Francis Crick Institute, 1 Midland Road, London NW1 1AT, UK; mark.skehel@crick.ac.uk; 8 Randall Centre for Cell and Molecular Biophysics, King’s College London, London WC2R 2LS, UK; franca.fraternali@gmail.com

**Keywords:** coenzyme A, CoAlation, thiolation, mixed-disulfide, CoA stabilization interactions, oxidative stress

## Abstract

Coenzyme A (CoA) is a key cellular metabolite known for its diverse functions in metabolism and regulation of gene expression. CoA was recently shown to play an important antioxidant role under various cellular stress conditions by forming a disulfide bond with proteins, termed CoAlation. Using anti-CoA antibodies and liquid chromatography tandem mass spectrometry (LC-MS/MS) methodologies, CoAlated proteins were identified from various organisms/tissues/cell-lines under stress conditions. In this study, we integrated currently known CoAlated proteins into mammalian and bacterial datasets (CoAlomes), resulting in a total of 2093 CoAlated proteins (2862 CoAlation sites). Functional classification of these proteins showed that CoAlation is widespread among proteins involved in cellular metabolism, stress response and protein synthesis. Using 35 published CoAlated protein structures, we studied the stabilization interactions of each CoA segment (adenosine diphosphate (ADP) moiety and pantetheine tail) within the microenvironment of the modified cysteines. Alternating polar-non-polar residues, positively charged residues and hydrophobic interactions mainly stabilize the pantetheine tail, phosphate groups and the ADP moiety, respectively. A flexible nature of CoA is observed in examined structures, allowing it to adapt its conformation through interactions with residues surrounding the CoAlation site. Based on these findings, we propose three modes of CoA binding to proteins. Overall, this study summarizes currently available knowledge on CoAlated proteins, their functional distribution and CoA–protein stabilization interactions.

## 1. Introduction

Reactive oxygen species (ROS) are cellular metabolites that participate in various signaling and metabolic pathways [1,2,3]. However, imbalanced levels of ROS can lead to oxidative stress and damage to cellular macromolecules: DNA, proteins and lipids [2,3]. Cells overcome oxidative stress by using different antioxidant systems, which include enzymes (superoxide dismutases, catalases, peroxiredoxins, glutathione peroxidases and methionine sulfoxide reductases), reducing pathways (thioredoxin/thioredoxin reductase, glutaredoxin/glutathione reductase pathways, bacilliredoxin/bacillithiol disulfide reductase pathways and mycoredoxin/mycothione reductase pathways) and low molecular weight (LMW) thiols (glutathione (GSH) and alternative thiols in microorganisms (bacillithiol (BSH) and mycothiol (MSH))) [4,5,6,7,8,9,10]. In recent years the antioxidant function of a key cellular metabolite, coenzyme A (CoA), was discovered [11,12,13,14]. CoA is an essential cofactor in all organisms and its biosynthesis involves enzymatic conjugation of cysteine, pantothenate (vitamin B5) and adenosine triphosphate (ATP) [15]. Similar to the antioxidant role of glutathione and protein glutathionylation (RS-SG), CoA protects protein cysteine thiols from hyperoxidation during oxidative stress by forming a mixed-disulfide bond, termed protein CoAlation (RS-SCoA) [11,12,16].

**Figure 1:**
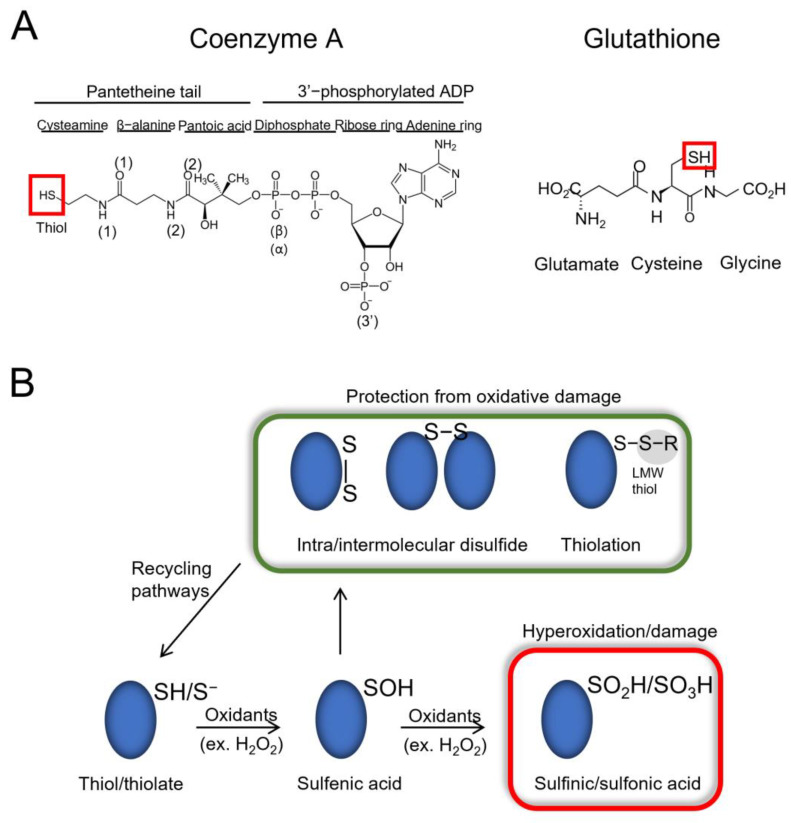
Overview of LMW thiols and protein thiolation. (**A**) Schematic structures of coenzyme A (CoA) and glutathione (GSH). The thiol groups are indicated with a red box. (**B**) In the presence of oxidants such as hydrogen peroxide (H_2_O_2_), proteins become reversibly oxidized, and can form a sulfenic acid (RSOH) on their cysteine residues. Elevated oxidant levels can lead to hyperoxidation of cysteine residues to sulfinic/sulfonic acids (RSO_2_H/RSO_3_H—red box), which can damage proteins and lead to loss of function. To overcome hyperoxidation, proteins can form intra/inter-molecular disulfide bonds, as well as mixed-disulfide bonds with low molecular weight (LMW) thiols (RSH—e.g., CoA, GSH), a process termed thiolation (e.g., CoAlation, glutathionylation). Protein thiolation can also result from thiol/disulfide exchange reactions (not shown). The presented modifications are involved in modulating the activity and subcellular localization of target proteins and protect them from irreversible oxidative damage. The modifications can then be reversed via reducing pathways.

Glutathione is a tripeptide (γ-glutamyl-cysteinyl-glycine) comprising three amino acids (cysteine, glutamic acid and glycine), whereas CoA is composed of a 3′-phosphorylated ADP moiety and a flexible pantetheine tail. Although structurally different, both molecules contain a reactive thiol group, allowing them to interact with a diverse range of substrates (Figure 1A). During oxidative stress, the physiological level of oxidants (e.g., hydrogen peroxide—H_2_O_2_) increases, leading to the oxidation of proteins at cysteine and methionine residues. The thiol group of cysteine residues can become oxidized to sulfenic acid (RSOH). A prolonged exposure to higher levels of oxidants can cause the hyperoxidation of cysteine residues to sulfinic acid (mostly irreversible modification) and even sulfonic acid (irreversible modification) [1,17]. When sulfenic acid is formed on a cysteine, LMW thiols (GSH, BSH, MSH and CoA) can perform a nucleophilic attack on the sulfur atom of the sulfenic acid and form a mixed-disulfide bond in a process termed thiolation (glutathionylation, bacillithiolation, mycothiolation and CoAlation). This prevents the hyperoxidation of the protein cysteine and in many cases, its irreversible loss of function [8,10,18,19,20,21] (Figure 1B). Protein thiolation could also occur through thiol-disulfide exchange reactions. Furthermore, sulfenic acids can also react with hydrogen sulfide to form persulfides, which are involved in physiological and pathological processes [22]. In the latter case of thiol-disulfide exchange reactions, there could be an interplay between persulfides and CoA. Overall, the formation of a mixed-disulfide bond with proteins can modulate the activity, structure, subcellular localization and regulatory interactions of modified proteins.

Over the past few years, research on protein CoAlation has shown that it is a widespread and reversible post-translational modification (PTM). Established cell lines and single-cell and multicellular organisms were shown to contain increased levels of CoAlated proteins upon exposure to oxidative or metabolic stress using a combination of anti-CoA monoclonal antibodies and LC-MS/MS (liquid chromatography tandem mass spectrometry) methodologies [11,12,23,24]. To date, CoAlation was found to modulate the activity of modified proteins, induce significant conformational changes and protect cysteine residues from hyperoxidation under oxidative stress. For example, the CoAlation of *S*. *aureus* and *Citobacter* sp. 5-77 glyceraldehyde-3-phosphate dehydrogenase (GAPDH) was shown to reversibly inhibit its activity [12,25], as previously demonstrated for glutathionylated (rabbit muscle GAPDH) and mycothiolated (*Corynebacterium diphtheriae* GAPDH) forms [26,27]. Furthermore, CoAlation of other proteins, such as aurora kinase A, peroxiredoxin 5 and metastasis suppressor protein NME1, were also shown to reversibly inhibit their function, mediated by covalent modification of their catalytic cysteines or cysteine residues located close to catalytic sites [28,29,30]. In the case of aurora kinase A, NME1 and GAPDH, the ADP moiety of CoA interferes with the binding of ATP or nicotinamide adenine dinucleotide (NADH), which inhibits their enzymatic activities. A study performed on the transcription factor accessory gene regulator AgrA (*S*. *aureus*) showed that CoAlation at its Cys199 interfered with its binding to DNA [31]. Using immunohistochemistry and in vitro studies together with the LC-MS/MS analysis, tau, a protein involved in the pathogenesis of Alzheimer’s disease, was also shown to be CoAlated [32]. Overall, these studies show the important cellular role of CoA in regulating the function of proteins and their downstream interacting partners under different cellular stress conditions.

Protein CoAlation and the antioxidant role of CoA is an emerging field of research, and unlike the well-studied roles of GSH and protein glutathionylation in redox regulation, the molecular mechanisms by which CoA regulates the function of modified proteins, their subcellular localization and the interaction with downstream partners in health and disease remain to be investigated. In this study, we combined known mammalian and bacterial CoAlated proteins (2093) and grouped them into two larger datasets: mammalian (1170) and bacterial (923) CoAlomes (CoAlated proteins). We performed functional classification of all CoAlated proteins, and showed that CoAlation is widespread among proteins implicated in cellular metabolism (59–60%), stress response (7–8%) and protein synthesis (7 and 17% for mammalian and bacterial proteins, respectively). By analyzing 35 published CoAlated protein structures, we determined three main types of CoAlated proteins, showed that CoA is a very flexible molecule (extended and bent forms) and characterized the specific CoA–protein stabilization interactions. Finally, we suggest three possible modes of protein CoAlation, based on the binding of the ADP moiety and the pantetheine tail, which mediate the specificity of covalent protein modification by CoA. Overall, our study summarizes all currently known CoAlated proteins, defines their functional distribution and expands our knowledge on the different types of CoAlated proteins and the possible modes of CoA binding to proteins.

## 2. Materials and Methods

### 2.1. Mammalian and Bacterial CoAlome (CoAlated Protein Dataset) Construction

To date, CoAlated proteins have been identified in mammalian cells and tissues, and bacteria exposed to oxidative stress using the developed liquid chromatography tandem mass spectrometry (LC-MS/MS)-based methodology. Generated CoAlated peptide sequences were combined to construct two datasets of mammalian and bacterial CoAlated peptides/proteins (Tables S1 and S2) [11,12,24]. To construct the larger mammalian CoAlome (CoAlated protein dataset) (Table S1), the CoAlated proteins from rat tissues and HEK293/Pank1β (human embryonic kidney 293, pantothenate Kinase β-overexpressing) cells were joined, and protein/peptide repeats were removed—this includes the protein isoforms (Table S1). A similar approach was used to construct the larger bacterial CoAlome (Table S2).

### 2.2. Functional Classification of Mammalian and Bacterial CoAlated Proteins

The functional classification of mammalian and bacterial CoAlated proteins was performed using the PANTHER Classification System [33] from PANTHER (Protein ANalysis THrough Evolutionary Relationship—version 16) [34]. The list of gene names of each protein from the CoAlation datasets was used as an input, taking into consideration the species type. The output from the software includes mammalian and bacterial functional annotation pie charts, where percentages of different cellular functional classifications (metabolic processes, protein synthesis, stress response and transport, among others) are presented. The gene entries not recognized by PANTHER were searched either manually using UniProt or using the functional annotation tool from DAVID (Database for Annotation, Visualization and Integrated Discovery) [35,36]. The subcellular localization of mammalian CoAlated proteins was determined using the functional annotation tool from DAVID.

### 2.3. Analysis of the Different Types of CoAlated Proteins

The protein data bank (PDB) was used to search for published protein structures containing CoA. A total of 612 structures were exported (on 31 July 2021) that contained either CoA or CoA derivatives (e.g., acetyl-CoA and phenyl-acetate-CoA). From the 612 structures, we selected those structures that contained CoA, and not its derivatives. We then used PyMol—a protein structure visualization tool, and manually selected 35 structures (Table S3) that have CoA disulfide bonded with a protein (CoAlated) or the CoA thiol group located <3 Å away from the protein cysteine residue. The different types of CoAlated proteins were selected following the manual observation of all 35 CoAlated structures. The localization (buried or solvent exposed) of the CoAlated cysteine residues of all 35 structures was investigated. To determine whether the CoAlated cysteine residue is solvent exposed, the solvent accessible surface area (ASA) of the CoAlated cysteine was calculated using PyMol. The following formula was used to determine the relative solvent accessibility (RSA) of the CoAlated cysteine: RSA = ASA/MaxASA, where Cys MaxASA = 167 Å [37]. RSA values above 20% indicated solvent exposed cysteine residues [38]. Following the analysis of the localization of the cysteines within the CoAlated protein structures, we also investigated whether CoA has stabilization interactions with other protein subunits, which would indicate that CoA can be stabilized by more than one protein subunit (Table S3).

### 2.4. Analysis of CoA’s Structural Flexibility and 3D Stabilization Interactions

Using the PyMol visualization tool, we focused on studying the CoA conformation for each of the 35 structures, which were either in an extended or bent form. The distances (Å) of CoA segments were measured using PyMol.

To determine the 3D stabilization interactions within CoAlated proteins, we studied the protein–ligand (CoA) interactions using LigPlot+ (version2.2.4) [39,40], where 2D protein–ligand interaction diagrams were obtained, and PyMol, where we visualized 3D stabilization interactions. The formation of stabilization interactions between CoA and protein residues were investigated taking into consideration the hydrogen bonds (2.6–3.3 Å), hydrophobic interactions (van der Waals interactions and π–π interactions) (3.3–4 Å) and salt bridges [41,42]. To understand which amino acid residues stabilize different CoA segments (ADP moiety and pantetheine tail), we manually summarized the interactions between CoA and the CoAlated cysteine microenvironment for all the 35 selected structures (Table S3). Then, we calculated the percentages of the different types of amino acids (positively/negatively charged, polar uncharged, hydrophobic aliphatic and hydrophobic aromatic) that stabilize each CoA segment. The types of amino acids surrounding the CoAlated cysteine residue were also reported.

### 2.5. Construction of Mammalian and Bacterial 7-Amino-Acid-Long Peptide Datasets and 2D WebLogos

For both the mammalian and bacterial CoAlated protein datasets, FASTA sequences were exported from UniProt. Using a C++ program, from all the CoAlated protein FASTA sequences, we were able to export 7-amino-acid-long peptides, which have their CoAlated cysteine residues at the center (position 0) and the surrounding amino acids at positions ±3.

With C++ programming, the letter “X” was introduced to complete vacant positions within the 7-amino-acid-long peptide sequences. The latter occurred in certain cases, where the CoAlated cysteine residues were located at the end of a protein sequence. The outcome was a non-redundant large dataset containing 7-amino-acid-long peptides, composed of a central CoAlated cysteine (position 0) and three flanking amino acids (±3).

WebLogo (version 2.8.2) [43] was used to obtain the 2D mammalian and bacterial CoAlation trends. The 7-amino-acid-long peptide datasets were used as input files (Section 2.3). The percentage frequencies of the type of amino acid per motif per position were calculated. The amino acid residue frequency at a certain position above 25% was considered significant [44]. The peptides that contained a residue belonging to a certain type of amino acid (positively or negatively charged, polar or non-polar) on a certain position on either side (±3) of the CoAlated cysteine (position 0) were selected and grouped into categories. From the outcome of the analysis, amino acid preferences were observed.

## 3. Results

### 3.1. Construction of Mammalian and Bacterial Datasets of CoAlated Proteins

The development of highly specific anti-CoA monoclonal antibodies and a reliable LC-MS/MS methodology allowed us to identify numerous CoAlated proteins in mammalian cells and tissues (HEK293/Pank1β cells, rat liver and heart) and bacterial cells (*Staphylococcus aureus*, *Bacillus subtilis* and *Bacillus megaterium*) subjected to oxidative or metabolic stress [11,12,24]. To have an overview of all currently known CoAlated proteins, we combined available LC-MS/MS results, and constructed the mammalian CoAlome (dataset) of 1170 proteins (Table S1) and the bacterial CoAlome (dataset) of 923 proteins (Table S2). Both datasets combined contain 2093 CoAlated proteins, with a total of 2862 sites of CoAlation (Table 1).

### 3.2. Functional Characterization of Mammalian CoAlated Proteins

**Figure 2:**
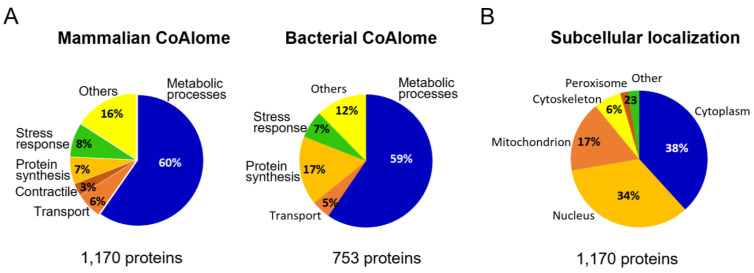
Functional analysis of CoAlated proteins and their subcellular localization. (**A**) Pie charts representing the molecular functions of mammalian and bacterial CoAlated proteins are shown. The percentages were calculated based on PANTHER (Protein ANalysis THrough Evolutionary Relationship—version 16) outputs. For both pie charts, metabolic processes represent the most predominant functional group of all CoAlated proteins. Important to note is that within the bacterial dataset (923 proteins), 170 proteins had unknown functions and therefore, only 753 proteins were used for the PANTHER analysis. (**B**) Pie chart representing the subcellular localization of mammalian CoAlated proteins. The segregation of CoAlated proteins using DAVID database revealed that the majority of CoAlated proteins are located within the cytoplasm, followed by the nucleus and mitochondria.

To have a better understanding of the protective and regulatory role CoA holds under cellular stress conditions, we classified the molecular function of mammalian and bacterial CoAlated proteins using the PANTHER Classification System (PANTHERv16) (Figure 2A). Out of the 1170 mammalian CoAlated proteins, the majority (60%) were involved in metabolic processes (Figure 2A). These included proteins involved in the TCA (tricarboxylic acid) cycle (malate dehydrogenase, citrate synthase, succinate dehydrogenase and isocitrate dehydrogenase), glycolysis (GAPDH), lipid metabolism (3-ketoacyl-CoA thiolase, acyl-CoA dehydrogenase and acetyl-CoA acetyltransferase) and amino acid metabolism (acyl-CoA synthetase and adenosylhomocysteinase), among others. The second largest group of functionally related proteins includes those involved in stress response (8%), such as methionine-*R*-sulfoxide reductase B2, thioredoxin, sulfiredoxin 1, thioredoxin reductase 2 and peroxiredoxins 1–6. Many proteins that participate or are associated with protein synthesis are also CoAlated (7%), including translation initiation factors EIF1 and EIF5, elongation factors 2–5, release factor ETF1, multiple ribosomal proteins of structural function (40S ribosomal protein S8 and 60S ribosomal protein L11) and key transcription factors (nuclear factor NFκβ, hepatoma-derived growth factor and metastasis-associated protein MTA2) (Figure 2A).

We explored the overall distribution of mammalian CoAlated proteins within the cell (1170 proteins—Figure 2B) and found that they are mainly localized in the cytoplasm (38%) and nucleus (34%), followed by the mitochondrion (17%). The reported total level of CoA in mitochondria ranges between 2 to 5 mM, which is much higher than the cytosolic and nuclear CoA levels, 0.05 to 0.14 mM [14,15]. There are 199 CoAlated mammalian mitochondrial proteins, which constitute 17.5% of the whole human mitochondrial proteome, reported to be consisting of 1136 proteins [45].

### 3.3. Molecular Function of Bacterial CoAlated Proteins

The bacterial dataset (923 proteins—Table S2) has 170 proteins with unknown functions and therefore, only 753 proteins were used to perform the PANTHER functional analysis (Figure 2A). The genome of *B. megaterium* is not well characterized, which limited the functional analysis of the 170 proteins. The functional distribution obtained was similar to the mammalian dataset, where proteins involved in metabolic processes comprised the majority of the CoAlated proteins (59%). This group includes proteins involved in the electron transport chain (respiratory nitrate reductase 1 beta chain and putative electron transfer flavoprotein subunit YdiR); TCA cycle (fumarate hydratase class II and succinate-CoA ligase α-subunit); and other key enzymes such as acetyl-CoA carboxylase carboxyl transferase β-subunit, lipid kinase and lactate dehydrogenase. Proteins involved in galactose metabolism (UDP-galactose 4-epimerase), as well as nitrogen fixation (nitrogen fixation protein NifU), were also among the CoAlated proteins. Proteins involved in stress response (7%) include major antioxidants, such as alkyl-hydroperoxide reductase C, various thiol peroxidases, thiol peroxidase-like proteins, bacillithiol biosynthesis deacetylase (BshB2) and methionine sulfoxide reductase B. Interesting to observe is the increase in CoAlated proteins within the “protein synthesis” classification of the bacterial dataset (17%) compared to the mammalian dataset (7%). This pool of CoAlated proteins includes structural components of the ribosome—50S ribosomal proteins L2, L32 and L14 and 30S ribosomal proteins S14 and S18; and proteins involved in translation, such as the translation initiation factor IF-3.

### 3.4. Structural Analysis Reveals Three Main Types of CoAlated Proteins

**Figure 3:**
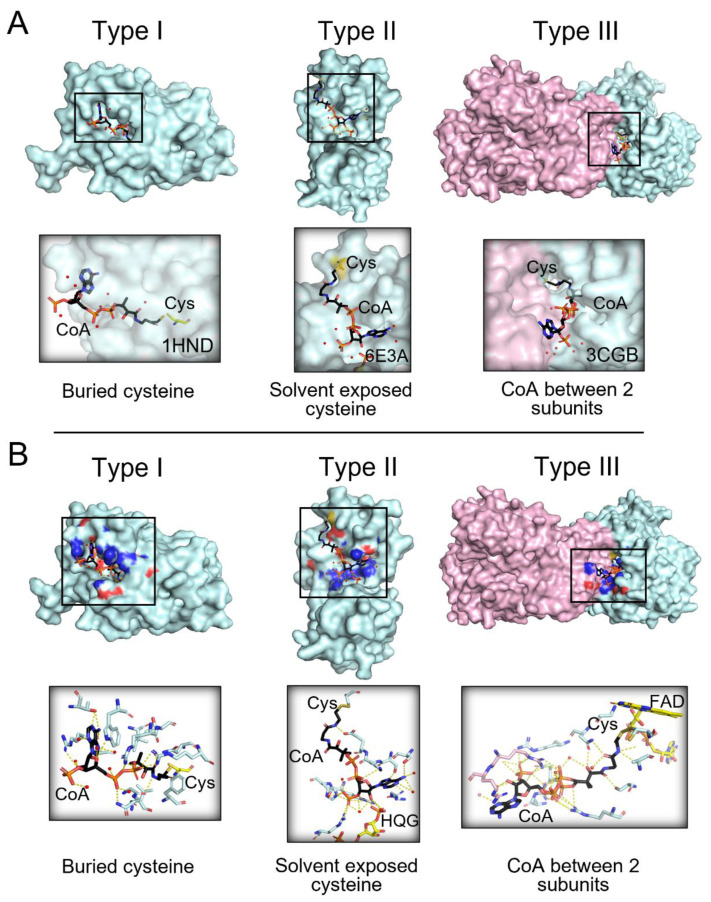
Three main types of CoA binding to modified proteins. (**A**) The three main types of binding to modified proteins are shown. In Type I (e.g., PDB: 1HND—beta-ketoacyl-acyl carrier protein synthase III), CoA is bound to a buried cysteine; in Type II (e.g., PDB: 6E3A—tRNA 2′-phosphotransferase), CoA is bound to solvent exposed cysteine; in Type III (e.g., PDB: 3CGB—CoA disulfide reductase homodimer), CoA is stabilized between two protein subunits. (**B**) Examples of each type of CoAlated protein and the CoA stabilizing interactions are shown. The CoAlated proteins are shown in cyan surface view. In Type III CoAlated proteins, the second protein subunit forming stabilizing interactions is indicated with a pink surface view. For the surface view presentation, the residues interacting with CoA are also shown, where blue represents the nitrogen atoms and red, the oxygen atoms. CoA is shown in a black stick conformation, and water molecules are shown in red balls. Yellow dashes indicate hydrogen bonding between CoA and protein residues and water molecules.

Understanding how CoAlation can regulate downstream signaling and the function of modified proteins is of great importance to further unravel its cellular antioxidant role. Therefore, we studied the site of CoAlation by searching for known 3D structures of CoAlated proteins from the Protein Data Bank (PDB). A total of 35 structures were observed to have either CoA covalently bound to the protein (CoAlated) or the CoA thiol group located less than 3 Å away from the protein cysteine thiol (Table S3). From the structures analyzed, we found three main types of CoA binding to proteins (Figure 3 and Figure S1). Categorization of the types of CoA binding focused on the localization of the CoAlated cysteine residues. In general, cysteines can be either buried within the protein structure or solvent exposed. In Type I CoAlated proteins, CoA interacts with a buried cysteine residue (11 of 35 structures) (Figure 3 and Table S1). In this case, the surface residues of the protein could potentially initiate the stabilization of the ADP moiety, leading to local structural changes that allow the pantetheine tail to gain access to the buried cysteine residue located within a defined groove/pocket. This allows the formation of a mixed-disulfide bond (Figure 3—Type I). On the other hand, in Type II CoAlated proteins, CoA interacts with a solvent exposed cysteine (10 of 35 structures). In this case, surface residues mainly stabilize both the ADP moiety and the pantetheine tail, which could potentially project the thiol group of CoA to the targeted Cys residue (Figure 3—Type II).

We also observed a third type, where CoA is localized within the interface of two functional domains/subunits of a protein (Figure 3 and Figure S1—Type III). It is interesting to observe that CoA can form stabilizing interactions with both domains/subunits, which target the pantetheine thiol group towards the modified Cys residue (Table S3). This indicates that the CoA binding pocket/interface can be created upon interaction of two protein subunits and their function could be regulated by CoAlation. Further experimental studies can reveal the role of CoA in regulating protein-protein interactions under various cellular conditions.

### 3.5. CoA Is a Bulky but Flexible Molecule When in Complex with Covalently Modified Proteins

**Figure 4:**
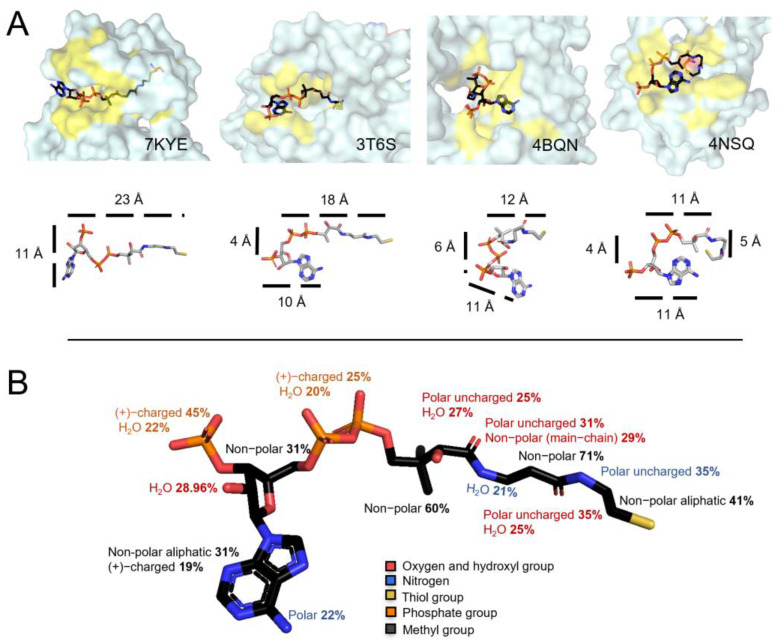
CoA flexibility and stabilization interactions. (**A**) Surface view of four structures showing CoA in different conformations. CoA is colored based on different elements: the Cys residue close to the CoA thiol group is shown in orange and the yellow color represents the location of residues forming stabilizing interactions with CoA. The second row focuses on the CoA structure flexibility and the distances (Å) of its segments. The CoA structure is observed to bend at the diphosphates or at different locations within the pantetheine tail. In an extended form, the CoA tail and the diphosphates are 23 Å, and in a bent form it can be 11 Å in one direction and 5 Å in another. (**B**) CoA–protein stabilization interactions in CoAlated proteins. The types of residues interacting with the different CoA segments are shown (Table S3). CoA is colored based on different elements; phosphate groups are orange, oxygen atoms and hydroxyl groups are red, amino groups are blue and the thiol group is yellow.

Compared to the well-studied LMW thiol glutathione (GSH), which is a tripeptide (γ-Glu-Cys-Gly), CoA is a bulkier molecule composed of a 3′-phosphorylated ADP moiety and a pantetheine tail, which has a reactive thiol group at its end (Figure 1A). The microenvironment of the protein cysteine, which becomes glutathionylated or CoAlated, plays an important role in establishing stabilizing interactions with GSH or CoA, leading to the formation of a mixed-disulfide bond (Figure 1B). By examining the 35 selected structures, CoA was observed to be either in an extended or a bent form (Figure 4A), as previously reported [46]. CoA can bend either at the diphosphates or at different locations within the pantetheine tail. Interestingly, CoA shows a very high structural flexibility (Figure 4A). For example, in an extended form, the CoA ADP moiety and the pantetheine tail are ~23 Å in length, while in an extreme bent form, the CoA tail can bend to ~11–12 Å in one direction and ~5 Å in another direction (Figure 4A). The distances were measured using PyMol. This analysis shows that CoA is a very flexible molecule capable of modifying its structure to establish stabilizing interactions within the environment of the CoAlated protein cysteine, whether the latter is solvent exposed or buried (more hydrophobic microenvironment) within the protein structure.

### 3.6. Profiling Coenzyme A–Protein Interactions and the Amino Acids Involved

We then investigated the different types of amino acid residues (non-polar aliphatic, non-polar aromatic, polar uncharged, and positively or negatively charged) interacting directly or via water molecules through their side chain or main chain with the different CoA segments (pantetheine tail and ADP moiety) (Figure 1A and Figure 4B—Table S3). Both PyMol and LigPlot+ were used to analyze the CoA–protein stabilization interactions (hydrogen bonding, salt bridges and hydrophobic interactions (Van der Waals interactions and π–π interactions)), and the results are summarized in Table S3 and Figure 4B.

The CoA pantetheine tail and the ADP moiety diphosphates (*α* and *β*) contain a sulfhydryl group (-SH), a hydroxyl group (-OH), methyl groups, amide groups (-NH) and phosphate groups (Figure 1A). Our analysis reveals that there are alternating polar uncharged and non-polar residues interacting with the CoA pantetheine tail (Figure 4B—Table S3). Within the CoA pantetheine tail, hydrogen bonding (H-bonding) is observed between the microenvironment of the protein cysteine and the polar groups (O, HO-, -NH) of CoA, while its methyl groups are stabilized by hydrophobic interactions. Water molecules also participate in the stabilization of the pantetheine tail through H-bonding. The cysteamine ethylene group (-CH_2_-CH_2_-) is mainly surrounded by non-polar aliphatic amino acids (41%), which form hydrophobic interactions (Figure 4B). The *α*- and *β*-phosphate groups (diphosphates) of the ADP moiety mainly interact with positively charged amino acids (Lys and Arg, 25%) through salt bridges and water molecules (20%) through H-bonding (Figure 4B). In some CoAlated protein structures, where CoA is in a bent conformation, stabilization interactions are observed within the different segments of CoA (Table S3).

The adenine ring (with an amino group) of the ADP moiety is mainly surrounded by a small hydrophobic patch and is stabilized by hydrophobic residues (31%) and/or π–π stacking interactions with aromatic residues (e.g., Tyr). The amino group (-NH_2_) of the adenine ring mainly interacts with polar residues (22%). The ribose ring (with a hydroxyl group and a 3′-phosphate) is mainly surrounded by non-polar residues (31%) and its hydroxyl group (-OH) mainly interacts with water molecules (29%). The 3′-phosphate group of the ribose ring interacts with positively charged residues (45%) through salt bridges, or water molecules (22%) through H-bonding (Figure 4B and Table S3). Overall, our findings show that CoA anchors itself to proteins by forming specific types of interactions during protein CoAlation.

One of the 35 structures analyzed showed CoA–ion interactions (eg. Mg^2+^ (PDB: 6I2U—Table S3). Within the structure of aurora kinase A (PDB: 6I2U), Mg^2+^ ions are observed to form salt bridges with phosphate groups of CoA (Figure S2—Table S3). The role of ions in regards to facilitating or impeding CoA binding to proteins during CoAlation remains to be investigated. Further structural-functional analysis can give us insights into the role of ions during CoAlation.

### 3.7. Exploring the Direct Microenvironment of the CoAlated Cysteine

We examined the microenvironment of the CoAlated Cys residues using the 35 selected structures (Table S3). Our results show that the most prevalent residues surrounding and interacting with the CoAlated cysteine are the non-polar aliphatic residues (mainly Leu (15/35 structures) and Ala (10/35 structures)) and polar uncharged residues (mainly Asn (10/35 structures)). Positively charged residues (His (7/35 structures) and Arg (4/35 structures)) that could contribute to the stabilization of the thiolate form (R-S-) of the Cys were also observed to stabilize or surround the CoAlated Cys. Negatively charged residues (Glu (6/35 structures)) are also present (Table S3). In addition to the 3D environment of the CoAlated Cys residues, amino acids located ±1–3 positions away from the CoAlated Cys could also play a role in protein CoAlation, whether it includes the stabilization of the protein Cys thiol or the CoA pantetheine tail. Therefore, we used the WebLogo analysis to determine the types of residues located ±1–3 positions from the CoAlated Cys; however, no specific pattern was extracted (Figure S3A). We expanded our 2D WebLogo analysis by using the mammalian and bacterial CoAlomes (Tables S1 and S2—Figure S3B,C). Based on the joined WebLogo analysis of mammalian and bacterial CoAlomes, we could only observe an enrichment of the Leu and Glu residues surrounding the CoAlated Cys residue (Figure S2C). Future structural and mechanistic studies of CoAlated proteins under physiological and stress conditions could further aid in the understanding of the role of these residues during protein CoAlation.

## 4. Discussion and Conclusions

The antioxidant system plays a vital role in protecting major cellular macromolecules during oxidative stress. Over the last decade, study on the antioxidant role of coenzyme A (CoA) and protein CoAlation has been emerging [13,14], where it is shown to be a widespread oxidative post-translational modification (PTM) that modulates protein structure and function [20,25,28,29,30]. Unlike the well-studied protein glutathionylation, the topic of protein CoAlation—focusing particularly on the site of CoAlation and the residues stabilizing CoA—is not well studied. Therefore, in this research, we focused on the detailed analysis of the specific CoA–protein stabilization interactions in order to advance our understanding of the redox regulation of proteins by CoA.

Our study showed that residues within the CoAlated cysteine environment contribute to the stabilization of both CoA and the CoAlated cysteine, facilitating CoAlation. Similar to CoA, glutathione (GSH—Figure 4A), mycothiol and bacillithiol are low molecular weight (LMW) thiols, which protect protein cysteines from hyperoxidation and are implicated in redox signaling [19,27,47,48]. Although research on the detailed site(s) of mycothiolation/bacillithiolation is not thoroughly discussed, protein glutathionylation on the other hand, is well studied [49]. Currently, there is a glutathionylation database (called DbGSH) that summarizes all experimentally proven glutathionylated proteins [50]. Two important features were considered when studying the site of glutathionylation, including the localization of the Cys residue and the *pKa* of its thiol group. The more solvent accessible the Cys residue, the more susceptible it is to glutathionylation (in most reported cases) [51]. The analysis of published structural studies revealed that CoA could form a disulfide bond not only with solvent accessible cysteine residues (Type II CoAlated proteins—Figure 3), but also with buried cysteines (Type I CoAlated proteins—Figure 3). As CoA is a very flexible molecule compared to GSH (Figure 1A and Figure 4A) and has a long pantetheine tail, which can interact with both polar and non-polar aliphatic residues, it can access buried cysteine residues by changing its overall conformation. H-bonding and polar interactions between a protein and a ligand have been reported to aid in the proper orientation of the compound within the protein structure. Once CoA binds to the protein, specific protein–CoA interactions could lead to local structural changes that allow the pantetheine tail to gain access to the buried Cys residue. Further molecular dynamics studies can expand our knowledge of the latter.

Studies on the glutathionylation site also showed the importance of the cysteine thiol *pKa* in facilitating glutathionylation [51]. The unperturbed protein cysteine residue has been reported to have a thiol *pKa* of around 8.5, which is higher than the cytosolic pH [52]. The reduction of the *pKa* value of protein cysteine thiols (R-SH) is required for the formation of a negatively charged and highly reactive thiolate (R-S^-^). Different factors could play an important role in facilitating the decrease in the thiol *pKa* to form the thiolate. These depend on the local microenvironment of the cysteine residue, including the H-bonding network, electrostatic stabilization interactions with cationic groups (Lys/Arg or protonated His side chains) and backbone (main-chain) peptide bonds, which have a permanent dipole moment and can donate an H-bond to stabilize the thiolate form [16,53,54,55,56]. Other factors include the localization of the cysteine residue at the N-terminal α-helix, where a cumulative helical macro-dipole or peptide bond dipoles may electrostatically stabilize thiol ionization [57,58,59,60].

At the site of cysteine glutathionylation, positively charged residues were reported to lower the *pKa* of the thiol and facilitate glutathionylation [49,61]. Using the 35 structures of CoA bound proteins, we found that the residues surrounding the CoAlated cysteine can stabilize the 3′-phosphorylated ADP moiety and the pantetheine tail, and those residues located within the direct microenvironment of the CoAlated cysteine (mainly non-polar aliphatic (e.g., Leu, Ala) and polar uncharged (e.g., Asn) residues) could be involved in the H-bonding network necessary to stabilize the cysteine thiolate group. Positively charged residues (His, Arg and Lys) within the direct microenvironment of the CoAlated cysteine and the CoA thiol group were also observed, similar to protein glutathionylation. These residues could potentially play a role in deprotonation and stabilization of the thiolate form of the cysteine. In addition to the *pKa* of the protein cysteine residue, catalytic residues that directly lower the *pKa* of the glutathione thiol have been described in glutathione *S*-transferases (GSTs), which are detoxifying enzymes [62]. The GST family contains enzymes with catalytic residues (e.g., Ser, Tyr or Cys), which deprotonate GSH and stabilize it via a H-bond network within the active site [63,64,65]. This allows GSH to be conjugated to different substrates including electrophiles, and in some cases such as GST-P1, to proteins [66]. Further experimental and computational studies are required to elaborate their function in protein CoAlation.

It is important to note that protein CoAlation could occur by different mechanisms depending on the type of cellular stress. For example, the presence of hydrogen peroxide (H_2_O_2_) could induce the formation of sulfenic acid on certain protein cysteine residues. CoA can then perform a nucleophilic attack on the sulfenic acid, leading to protein CoAlation (Figure 1B). In this case, the cysteine residue should have a lower p*K*a to facilitate CoAlation, and the residues within its microenvironment can contribute to the deprotonation of the cysteine thiol (R-SH) and/or the stabilization of the thiolate form (R-S^−^) of the cysteine side chain. On the other hand, during diamide stress, diamide induces protein disulfide bond formation, leading to protein CoAlation. In this case, the p*K*a of the cysteine thiol group does not need to be low. Therefore, depending on the type of stress, different residues could be involved in the process of protein CoAlation. To the best of our knowledge, the 35 structures studied in this paper were not performed under certain stress conditions (using stress agents). Therefore, future studies focusing on the CoAlation of proteins under different stress conditions could significantly aid in the study of the site of CoAlation and respective residues involved, such as those involved in the deprotonation of the cysteine thiol (R-SH), the H-bonding network required to stabilize the thiolate form (R-S^−^) and stabilization of the CoAlated Cys.

In certain proteins, the presence of a nearby cysteine residue could compete with CoA for the formation of either an intramolecular disulfide bond between the two cysteines or a CoA-cysteine mixed-disulfide bond, respectively. This could depend on the presence of different oxidizing agents and the microenvironment of the cysteine residue. Therefore, the presence of the second cysteine within the vicinity could play a role in determining the oxidation state of the protein (CoAlated or intramolecular disulfide bonded). We analyzed the 35 CoAlated structures and measured the distance between CoAlated cysteine and other cysteine residues located within its vicinity (data not shown). In these structures, the nearest cysteine residue is located 8.3 Å away from the site of Coalition, which is too far for a second disulfide exchange reaction to occur. Further bioinformatics studies are required to investigate the location of nearby cysteine residues relative to the sites of CoAlation in mammalian and bacterial CoAlomes. It would also be interesting to know whether cysteines involved in regulatory intramolecular disulfide bond formation are CoAlated under oxidative stress.

**Figure 5:**
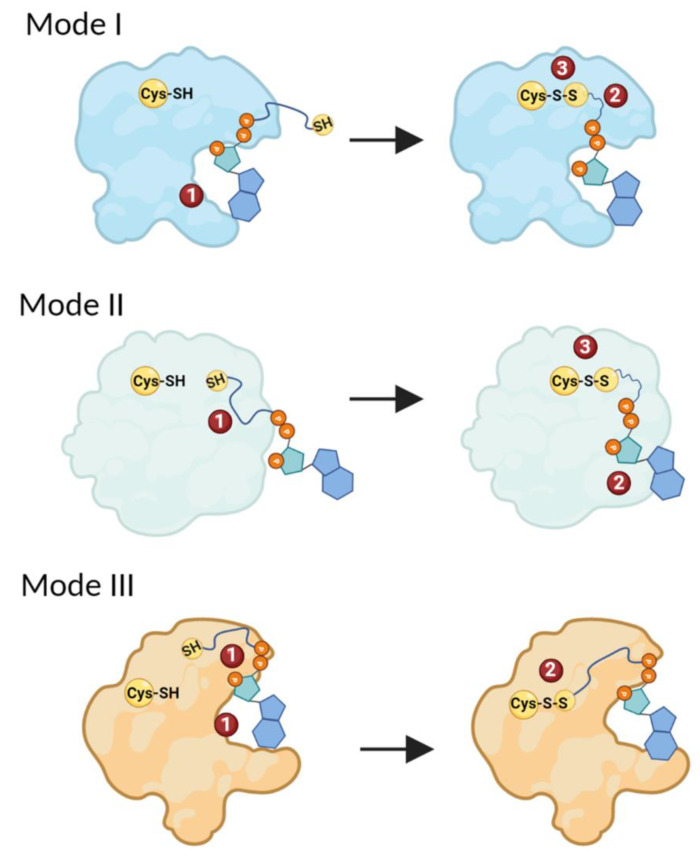
Different modes of CoA binding to proteins. In Mode I, the ADP moiety of CoA binds to a protein first. This primes the stabilization of the pantetheine tail, followed by the formation of a mixed-disulfide bond. In Mode II, protein CoAlation is initiated by the stabilization of the pantetheine tail of CoA, which primes the binding and stabilization of the ADP moiety of CoA and the formation of a mixed disulfide. In Mode III, both the ADP moiety and the pantetheine tail simultaneously bind the protein, followed by the formation of a mixed-disulfide bond. The different steps of CoAlation are indicated with numbers. The figure was generated using Biorender.

Based on the current available literature and the CoA–protein stabilization interactions analyzed in this study, we suggest three main possible modes of CoA binding to proteins (Figure 5). Within the first mode of CoA binding, protein CoAlation is initiated by the stabilization of the 3′-phosphorylated ADP moiety of CoA. This stabilization occurs within a hydrophobic patch composed of non-polar aliphatic amino acids, and/or aliphatic aromatic amino acids, which can stabilize the adenine ring of the ADP moiety by π–π interactions. Binding of the ADP moiety can lead to local structural changes that prime the pantetheine tail to gain access to the protein thiol group, whether it is located in a deep catalytic pocket (buried) or solvent exposed, and form a disulfide bond (Figure 5—Mode I). The structural-functional analysis of CoAlated aurora kinase A shows that CoA binds to the protein using the proposed mode, where the CoA ADP moiety binds to the aurora kinase A first, priming the stabilizing of the pantetheine tail and disulfide bond formation [28].

Many proteins from mammalian and bacterial CoAlomes contain the Rossmann fold, a structural feature found in nucleotide-binding proteins [67] that can facilitate the binding and stabilization of the ADP moiety of CoA if a cysteine residue is located within the vicinity of the Rossmann fold. Depending on the function of the Rossmann-fold-containing protein and localization of the CoAlated cysteine, CoA could either bind to the nucleotide-binding site (e.g., *Thermococcus kodakarensis* 2-dehydropantoate 2-reductase (PDB: 5AYV—Figure S4A–C)) or to a distinct location within the protein (e.g., *Thermus thermophilus* NADH-dependent coenzyme A disulfide reductase (PDB: 6RVB—Figure S4D)). By overlaying the 2-dehydropantoate 2-reductase NADP- and CoA-bound structures, we observed CoA to be bound to the NADP binding site. However, the orientations of the CoA adenosine/ribose rings are different from the 2′-phosphorylated NADP ring (Figure S4A–C). NADP 2′-phosphate (Arg31) and CoA 3′-phosphate (Arg257, Lys74, and Ser10) have different stabilization interactions. This analysis revealed that binding/stabilization of CoA to the nucleotide-binding pocket differs from that of NADP. CoA binding is most likely influenced by the localization of CoAlated cysteine and the presence of 3′-phosphate. Further bioinformatics, molecular dynamics and structural analysis of CoA-bound Rossmann-fold-containing proteins could shed light on the mode of CoA binding to these sites in comparison to NADP/NAD.

Within some of the analyzed structures, we observed strong stabilization interactions through CoA diphosphates, compared to other segments (Table S3). These stabilization interactions could guide the CoA pantetheine tail thiol group towards the protein cysteine residue for disulfide bond formation. Recently, structure-function analysis of human NME1/NME2 revealed that long-chain fatty acyl-CoA (LCFA-CoA), a CoA derivative, could inhibit the catalytic activity of these enzymes [68]. By mutating an Arg residue to a Glu in both NME1/NME2, the enzymes maintained their catalytic activity in the presence of LCFA-CoA. Further structural analysis of NME2 in complex with LCFA-CoA revealed that Arg plays an important role in stabilizing the diphosphates of CoA, and its mutation possibly prevents binding of the CoA derivative, and thus the enzyme maintains its activity in the presence of LCFA-CoA [68]. This shows the importance of the positively charged Arg residue in stabilizing CoA via interactions with the diphosphates. In our CoA stabilization interaction analysis, we observed the diphosphates to be stabilized mainly by positively charged residues and water molecules (Figure 4B—Table S3). The 3′-phosphate of the ADP moiety could also play a role in initiating specific CoA binding, but further structural and mutagenesis studies could aid in understanding its role.

The second suggested mode of CoAlation involves the binding and stabilization of the CoA pantetheine tail first. This would prime the binding of the ADP moiety followed by disulfide bond formation (Figure 5—Mode II). Residues located close to the CoA binding site play an important role in correctly orienting the pantetheine tail in order to gain access to the protein Cys residue. Further experimental and structural studies could shed light on the latter mode of CoA binding to proteins. The third suggested mode of CoAlation involves the simultaneous binding and stabilization of both the CoA ADP moiety and the pantetheine tail. The stabilizing interactions of both CoA segments are important for the correct positioning of the pantetheine tail, which allows the tail to gain access to the protein Cys residue and form a disulfide bond. Structural-functional analysis shows that *S. aureus* GAPDH uses Mode III for CoAlation [12].

Protein CoAlation is a widespread oxidative PTM, and its protective regulatory role under cellular stress conditions is vital in order to maintain the function of proteins involved in important cellular processes (metabolism, stress response and protein synthesis). Whether Cys residues are located on the surface of these proteins or they are buried within the structure, under cellular stress conditions, proteins undergo conformational changes and can be protected by CoA. This study summarizes all known CoAlated proteins from both bacterial and mammalian CoAlomes, and highlights their functional distribution within cells. By studying CoAlated protein structures, we defined the interactions CoA forms within the environment of the CoAlated Cys residue. These stabilizing interactions are protein specific and can prime the pantetheine tail to gain access to the protein Cys. We also defined the direct microenvironment of the CoAlated Cys, which could facilitate CoAlation. Based on the current study and available literature data, we suggest three possible modes of protein CoAlation. This study advances our knowledge of protein CoAlation and specifically, the site of CoAlation. Overall, our data could be used to estimate specific CoAlation sites on proteins with known 3D structures or homology models generated by AlphaFold. These will open doors to further understanding of the redox regulation of proteins by a key metabolic cofactor, CoA.

## Figures and Tables

**Table 1:** Summary of currently available mammalian and bacterial CoAlated protein datasets. The organism, cell or tissue type, and the number of total CoAlated proteins (peptides) for each of the mammalian and bacterial smaller datasets are shown. CoAlated protein isoforms were removed in order to calculate the total number of unique CoAlated proteins for each of the mammalian and bacterial datasets.

**Mammalian CoAlome**			
**Dataset**	**Organism, Cell or Tissue Type**	**CoAlated Proteins (Peptides)**	**Total Proteins (Peptides)**
**1**	*R. norvegicus* perfused heart [11]	26 (64)	1170 (1728)
	*R. norvegicus* liver mitochondria [11]	18 (30)	
	HEK293/Pank1β cells	1126 (1634)	
**Bacterial CoAlome**			
**Dataset**	**Organism, Cell or Tissue Type**	**CoAlated Proteins (Peptides)**	**Total Proteins (Peptides)**
**2**	*B. megaterium* [24]	355 (439)	923 (1134)
	*S. aureus* [12]	362 (448)	
	*B. subtilis*	206 (247)	
**Total CoAlated Proteins (Peptides) = 2093 (2862)**			

## Data Availability

Data is contained within the article or Supplementary Material.

## Supplementary Materials

The following supporting information can be downloaded at: https://www.mdpi.com/article/10.3390/antiox11071362/s1, Figure S1: Three different types of protein CoAlation; Figure S2: CoA-Mg^2+^ interactions; Figure S3: CoAlation site WebLogo representation; Figure S4: Analysis of CoA bound Rossmann-fold containing proteins; Table S1: Larger dataset of CoAlated mammalian proteins (CoAlome) and peptides; Table S2: Larger dataset of CoAlated bacterial proteins (CoAlome) and peptides; Table S3: Analysis of CoA-protein stabilization interactions using 35 published structures Containing CoA (CoAlated or CoA < 3 Å away from Cys).
