# Supplementary figures and images for: Profiling the Site of Protein CoAlation and Coenzyme A Stabilization Interactions
Source: Antioxidants (Basel). 2022 Jul 14;11(7):1362. doi: 10.3390/antiox11071362 (PMC9312308; doi:10.3390/antiox11071362)

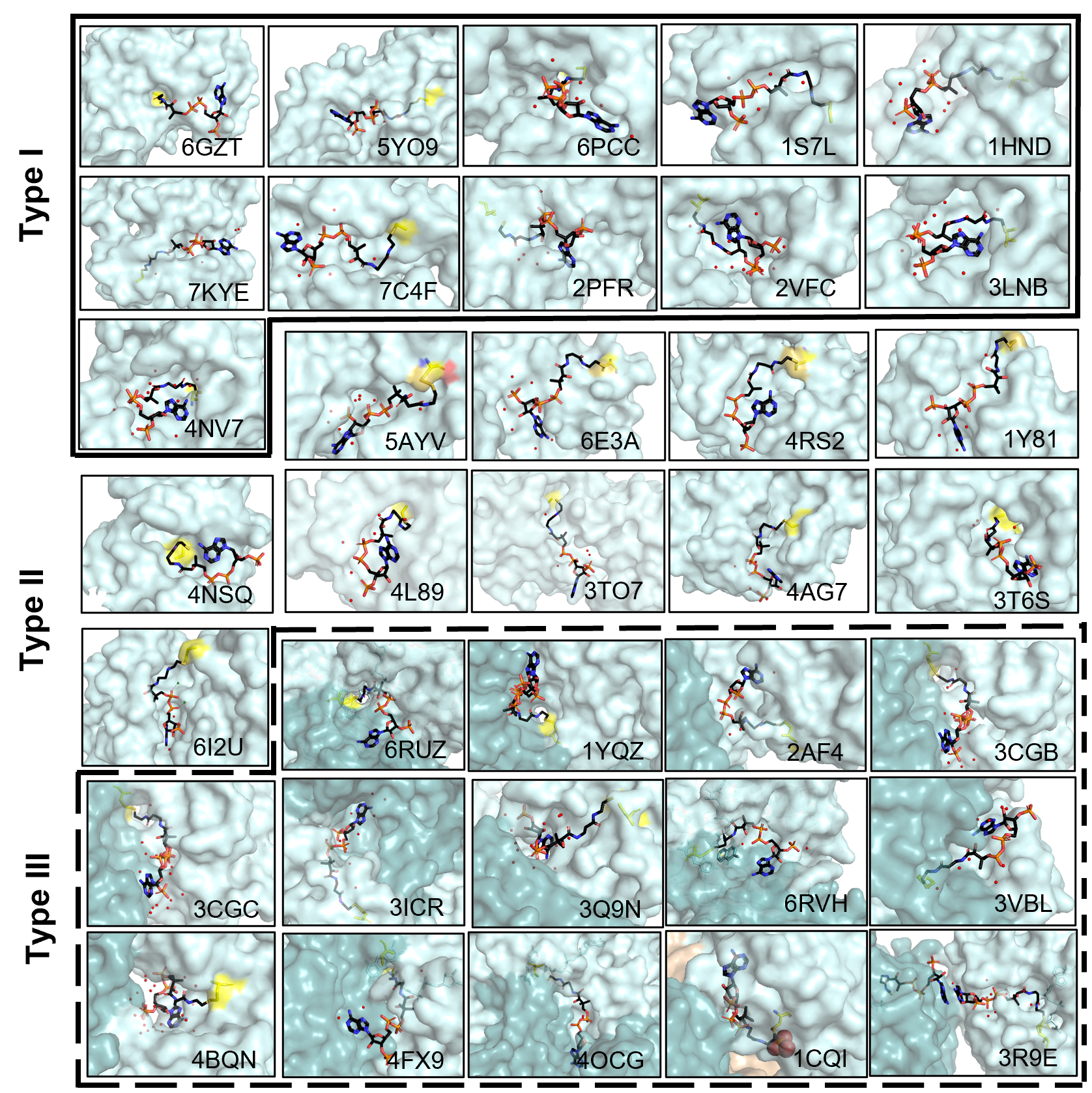

Supplement: Supplementary file 1 [file antioxidants-11-01362-s001.zip › FigureS1-300.tiff]

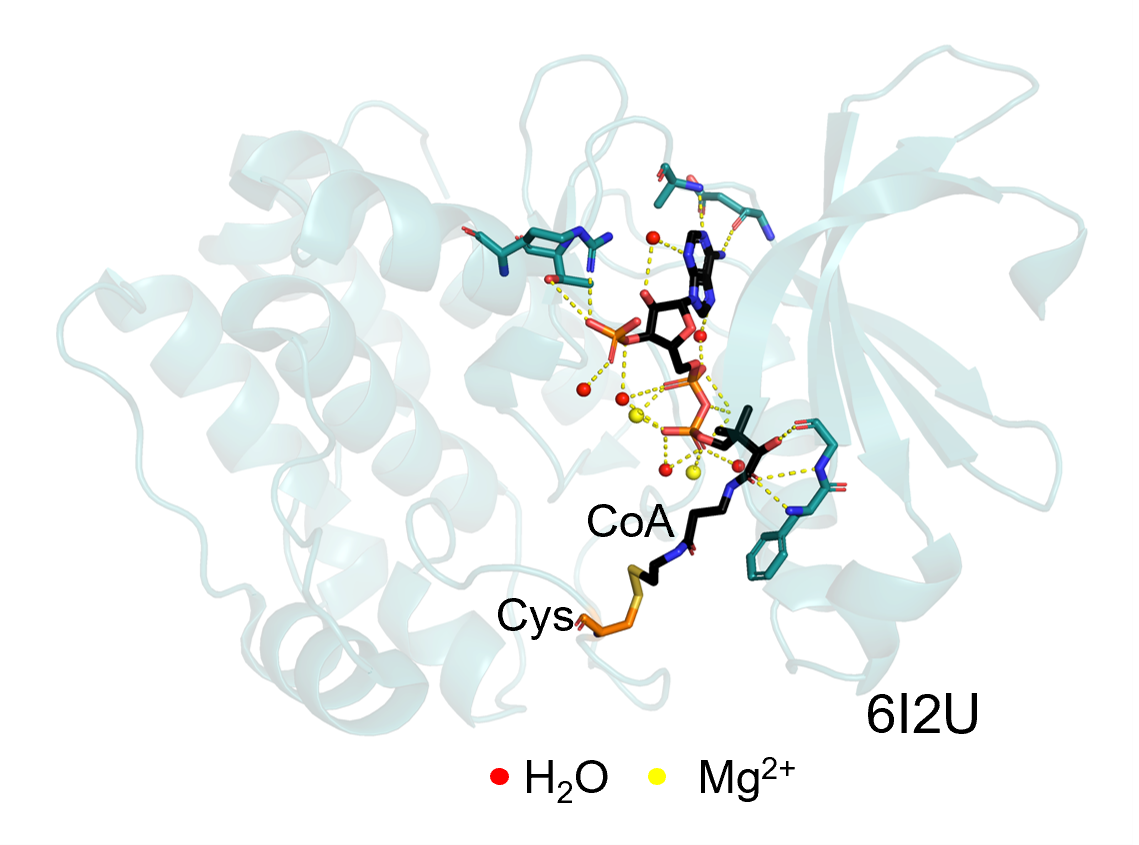

Supplement: Supplementary file 1 [file antioxidants-11-01362-s001.zip › FigureS2-300.tiff]

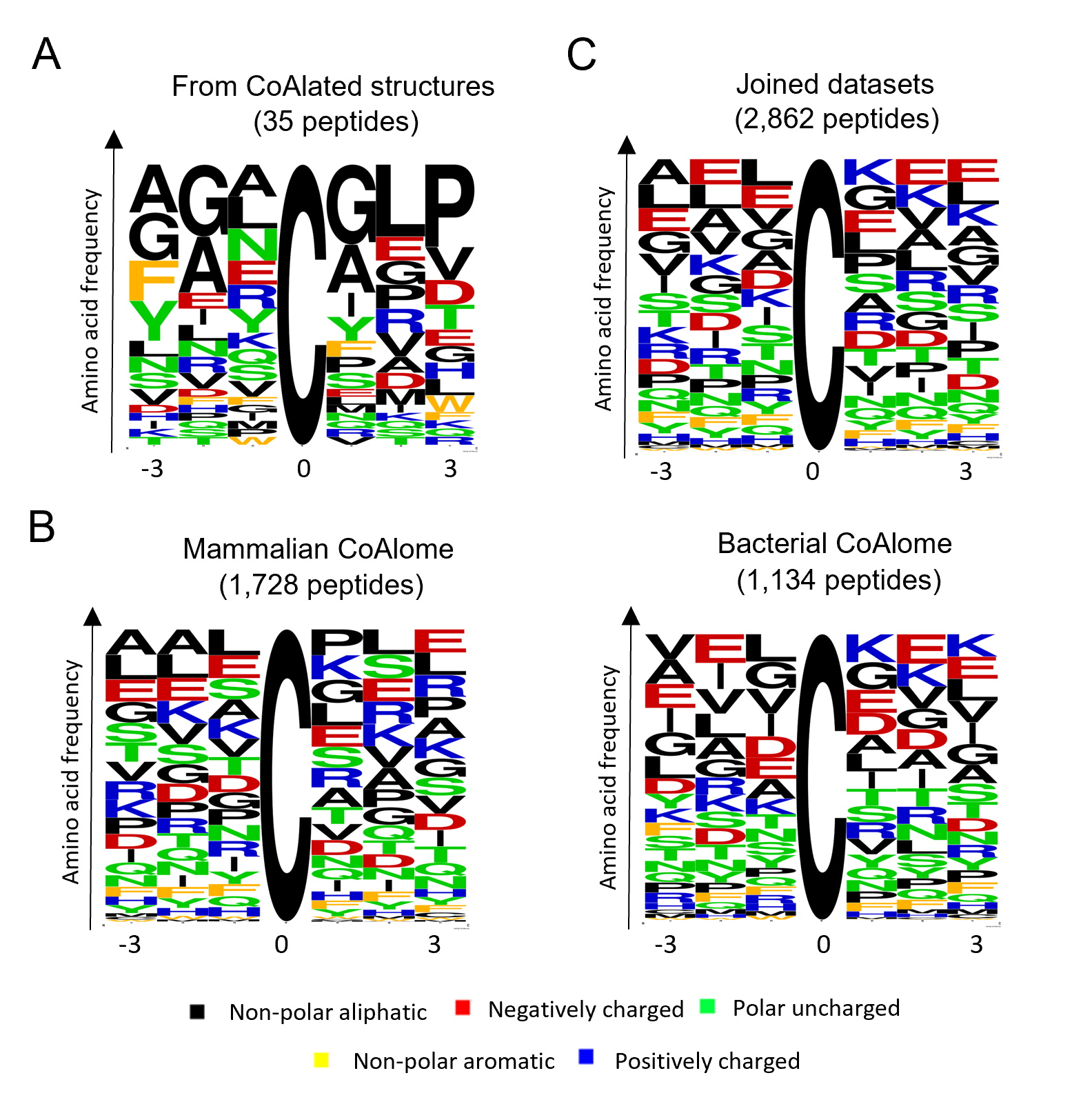

Supplement: Supplementary file 1 [file antioxidants-11-01362-s001.zip › FigureS3-300.tiff]

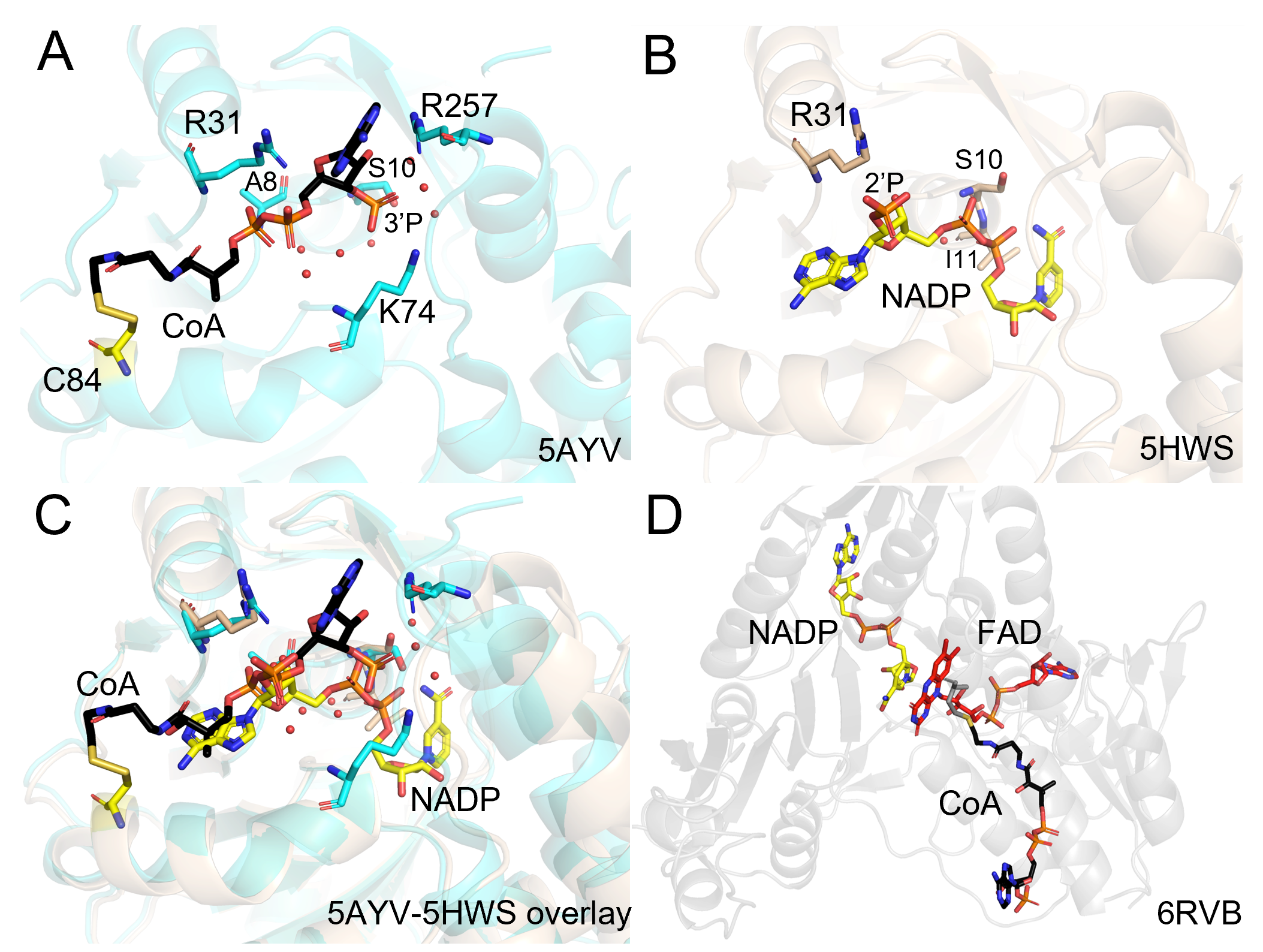

Supplement: Supplementary file 1 [file antioxidants-11-01362-s001.zip › FigureS4.tif]
